# Safety Assessment of High- and Low-Molecular-Weight Hyaluronans (Profhilo®) as Derived from Worldwide Postmarketing Data

**DOI:** 10.1155/2020/8159047

**Published:** 2020-06-20

**Authors:** Daniel Cassuto, Mara Delledonne, Giovanna Zaccaria, Immacolata Illiano, Andrea Maria Giori, Gilberto Bellia

**Affiliations:** ^1^Private Practice, Jerusalem, Israel; ^2^Private Practice, Milan, Italy; ^3^IBSA Farmaceutici Italia Srl, Lodi, Italy; ^4^IBSA Institut Biochimique SA, Lugano, Switzerland

## Abstract

**Background:**

At present, dermal fillers based on hyaluronic acid (HA) represent the most popular intervention of dermoesthetic medicine for the treatment of skin aging. Recent studies have shown that the combination of HA chains of different lengths and molecular weights improves tissue repair and regeneration through a synergistic mechanism. Profhilo® is a product available that has been on the market since 2015 and is based on stable, hybrid, and cooperative complexes (HyCoCos) produced by means of NAHYCO® Hybrid Technology, which is an innovative thermal process that rules out the use of any chemical reagents. The result is a filler with high biocompatibility and low viscosity that favors optimal diffusion at the tissue level to obtain the target bioremodeling of the facial contour. The objective of this review is to provide data from the overall postmarketing experience after 3 years of use and more than 40,000 patients treated with the medical device.

**Methods:**

All spontaneous postmarketing adverse event (AE) reports received from physicians and healthcare professionals worldwide between February 9, 2015, and February 8, 2018, associated with the use of the studied medical device and sent to the IBSA global safety database were analyzed.

**Results:**

In total, 12 adverse event reports were logged in the global database, and none were considered serious. Early-onset injection site reactions, i.e., swelling, edema, redness, ecchymosis, and erythema, were the most frequently observed. Late-onset local reactions (e.g., swelling, nodules) followed. The genesis of these reactions was considered, both by the reporting physician and IBSA, as being local reactions of hypersensitivity and/or due to inappropriate injection techniques. In no case was the product held liable for direct damage. All events resolved without any complications according to the treatment guidelines. Two late-onset reactions were collected.

**Conclusions:**

Although underreporting of minor events cannot be ruled out, the overall number of reports is very low, thereby supporting the high tolerability and safety of the product. After 3 years of postmarketing experience, the safety profile of the studied medical device is favorable and consistent with the product information.

## 1. Introduction

Minimally invasive procedures have revolutionized the treatment paradigm for both facial and body rejuvenation and the recent history of cosmetic surgery. Initially, developed exclusively for the treatment of fine lines and wrinkles, the concept of dermal fillers has expanded to include the correction of volume loss in the aging face, as well as improvement of damaged and scarring tissues, and has contributed to the increasing success of cosmetic surgery. The ideal soft tissue filler is effective, nonimmunogenic, nontoxic, noncarcinogenic, nonmigratory, easily applied, nonpalpable, painless, and long lasting [1]. Moreover, it should be low cost, provide lasting results and, while showing an acceptable persistence, ought to be easy to remove if necessary. Hyaluronic acid dermal fillers have most of these ideal characteristics [2].

As a linear polysaccharide composed of repeated disaccharide units of glucuronic acid and N-acetylglucosamine, hyaluronic acid (HA) forms an integral part of the natural extracellular matrix and is found in high amounts in several connective tissues, including the skin, vitreous humor of the eye and synovial fluid [3]. Due to its hygroscopic property, biocompatibility, and reversibility, HA is currently the most popular dermal filler used to replace volume loss due to aging. Therefore, over the past decades, various forms of HA fillers have been developed, each having different characteristics, such as the type and degree of crosslinking, gel viscosity, gel hardness, gel consistency, extrusion force, total HA concentration, and duration of presence in the skin [4]

A role for HA chains of different lengths has been reported in wound repair, especially considering the simultaneous occurrence of both high- (H-HA) and low- (L-HA) molecular-weight hyaluronan at an injury site in vivo. The effect of H-HA, L-HA, and the HHA/L-HA HyCoCos on wound closure was tested in keratinocyte cell monolayers, where these compounds provided faster regeneration and wound closure that was achieved in half the time of H-HA stimulated-cells and 2 [5] fold faster than the control. The outcomes of this research showed that, at both high and low concentrations, hybrid complexes performed better than HA alone, thus suggesting their potential as medical devices in both esthetic and regenerative medicine [5]. In the US, esthetic procedures with HA dermal fillers were rated as the second most popular nonsurgical procedure in 2017 by The American Society for Esthetic Plastic Surgery, and statistics worldwide confirm its medical use globally as well as the dramatic expansion of this market [6].

The advantages of HA dermal fillers are their ease of administration and rapid achievement of the desired esthetic improvement. When correctly performed, the safety profile of hyaluronic acid fillers is favorable, and the injection procedure is relatively safe [7].

The experience gained to date shows that the frequency of AEs is currently relatively low compared to other kinds of dermal fillers. However, other rare AEs can lead to severe complications requiring monitoring, early detection, and treatment [8, 9].

As the usage of HA dermal fillers is increasing due to expanded indications and types of procedures, the use of larger volumes and layering techniques, new classes of products, and repeated treatment will most likely result in an increase in the number of complications, even with an experienced physician.

Many factors may lead to AEs after dermal injection with hyaluronans [10–17]:

- HA is obtained from the fermentation of bacteria, which may be a source of impurities
- The breakdown products of HA crosslinked fillers in vivo could likewise elicit hypersensitivity reactions
- Differences in water-binding capacity among products could be relevant to localized reactions such as pain and swelling
- Patient history and anatomical characteristics represent predisposing factors in the occurrence of AEs
- Complications may be the consequence of relevant product-related factors, such as the concentration and rheologic properties of the filler, as well as the manufacturing processes (e.g., purification)
- A key role is played by the clinician, who has full control over the injection technique, as well as procedure-related factors, specifically, the depth, volume, speed, and accuracy of the injection. HA filler complications can be divided into early (which typically appear within hours to days post procedure) and delayed onset complications (which usually develop weeks to years post HA filler injection), as shown in Table 1
- The most common side effects associated with HA injection are site reactions, including edema, pain, erythema, itching, bleeding, and ecchymosis, which normally last less than one week
- Placing HA fillers too superficially might result in the so-called Tyndall effect, which appears as a bluish discoloration of the skin
- Displacement of the filler material is mainly a consequence of wrong technique or lack of experience of the physician and can lead to the early appearance of lumps, asymmetries and deformities
- Hypersensitivity reactions to HA injections reported since the 1990s are now believed to have been related to protein contaminants that were present in the first-generation HA preparations. As a matter of fact, the introduction on the market of a hyaluronic raw material with a protein content that is six times lower than the raw material previously used has led to a reduction in the frequency of hypersensitivity reactions
- Infections can result from the breach in skin surface integrity, and they can range from reactivation of herpes simplex infection to the formation of an abscess and cellulitis, which may be caused by the late-onset formation of a biofilm, which is a collection of bacteria surrounded by a protective and adhesive matrix. Bacteria present in biofilms use the implanted filler as a surface on which to attach and excrete their own matrix, which gives them the ability to survive, develop and resist antibiotic treatment. Long-lasting biofilms can eventually lead to tissue fibrosis
- Foreign body granuloma is a chronic inflammatory reaction that entraps an alien element into the body, preventing its migration and/or elimination. Even small amounts of residual protein contaminants, after HA filler purification, potentially carry a risk for hypersensitivity reactions and formation of granulomas. The incidence of foreign body granuloma formation after the injection of HA fillers ranges from 0.02% to 0.4%;
- The most severe complication regarding filler injections is vascular occlusion due to unintentional intravascular injection or embolization. Some anatomical areas, such as the glabella, alar base, nose, and temple, are known to be associated with higher risks for vascular complications

### 1.1. Profhilo®

Launched in 2015, Profhilo® is a novel HA preparation by IBSA that is based on stable hybrid cooperative complexes (HyCoCos), which is the first product developed by NAHYCO® Hybrid Technology, an innovative thermal production process patented by IBSA.

Package includes 1 prefilled syringe with 2 needles 29 G × ½ (0.33 x 12 mm) in the following available volume:−2 ml prefilled syringe − 32 mg (H − HA) + 32 mg (L − HA) of hyaluronic acid sodium salt in 2 ml of buffered sodium chloride physiological solution. The prefilled syringes are sterilized by moist heat, and the needles are sterilized with ethylene oxide.

The production process starts with a simple mixture of 32 mg of high-molecular-weight HA (1100-1400 kDa) and 32 mg of low-molecular-weight HA (80-100 kDa). The mixture is then stabilized by the abovementioned thermal process, which does not use crosslinking agents and consists of, first, a high-temperature step, followed by a low-temperature step.

The result is a product that boosts both remodeling and repair processes of tissues, even when scarring has occurred. It also improves skin laxity of the face, neck, and body.

PROFHILO® is indicated for the treatment of the face and body, in particular for the treatment of the malar-zygomatic and submalar areas. An initial cycle of two sessions at 30-day intervals is recommended, followed if necessary by maintenance procedures every 2 months. However, it is suggested to evaluate the specific PROFHILO® protocol according to the patients' degree of aging.

The product shows unique characteristics, such as a high HA concentration (64 mg/2 mL), ideal manageability, optimal tissue diffusion and low viscosity, and with a predominance of fluidity over elasticity (tan delta > 1). Moreover, several features provide HyCoCos with a high biocompatibility profile. First, HyCoCos are produced by means of the biosynthesis of a natural substrate without any further chemical modification; second, their thermally stabilized natural HA has a duration similar to that of a weak crosslinked gel. Compared to other native HA formulations, HyCoCos show the potential for a more effective global bioremodeling performance, and the simultaneous presence of high- and low-molecular-weight hyaluronan makes it a medical device that can be used in both esthetic and regenerative medicine. As proven by the research carried out by D'Agostino et al., the hybrid complex formed by H-HA and L-HA promotes wound healing of human keratinocytes *in vitro* better than HA alone.^5^ Moreover, this particular combination increases the expression levels of type I and type III collagens as well as elastin [18]. Finally, Stellavato et al. proved that HyCoCos enhance adipogenic differentiation and proliferation of adipose-derived stem cells (ASCs), which are used for recovery from local tissue ischemia and scar remodeling and potentially improve fat tissue renewal [19].

Over the last 3 years, these *in vitro* data have been validated and integrated by clinical studies, which have confirmed both the efficacy and tolerability of the studied medical device [20–23].

*In vitro* and clinical trials have confirmed the innovative characteristics of the product. Therefore, after 3 years of marketing use, the manufacturer has reviewed the postmarketing safety data to provide a full and extensive description of the product profile.

### 1.2. Injection Technique

IBSA recommends 2 sessions with a one-month interval by means of the BAP (Bio Esthetic Points) Techniques to minimize the risks and maximize the product's flowability.

Five points for intradermal administration of PROFHILO® must be identified as follows:

1. *Zygomatic Protrusion.* It is recommended to stay at least 2 cm away from the lateral canthus (external corner) of the eye.
2. *Nasal Base.* A line connecting the nostril and tragus and a perpendicular line starting from the pupil must be drawn to locate the injection point at the intersection of the 2 lines.
3. *Tragus.* It is recommended to stay at least 1 cm anterior to the inferior margin of the tragus.
4. *Chin.* A vertical line at the center of the chin and a perpendicular line one-third of the distance from the top of the vertical line have to be drawn. From the point of intersection, it is indicated to move 1.5 cm towards the oral commissure to locate the injection point.
5. *Mandibular angle.* The fifth point is located 1 cm above the mandibular angle.

Then, 0.2 ml of product with the bolus technique must be injected in the deep dermal/subcutaneous levels.

Injections should be followed by a gentle massage.

## 2. Methods

From February 9, 2015, to February 8, 2018, IBSA Farmaceutici Italia received spontaneous reports from physicians who used the studied medical device on their patients. The safety data were evaluated on an annual basis in relationship with sales data for the product worldwide. Therefore, three periods were identified for the analysis: February 9, 2015-February 8, 2016; February 9, 2016-February 8, 2017; and February 9, 2017-February 8, 2018.

Furthermore, all the adverse events were assessed based on their time to onset from the injection:

(i) Early onset, including
  - immediate onset-within 24 hours
  - delayed onset: <=72 hours
(ii) Late onset: >72 hours

As reported in the product information, the current posology for the use of the medical device is “an initial cycle of two treatment sessions at 30-day intervals, followed if necessary by maintenance treatments every 2 months. However, it is suggested to evaluate the specific Profhilo® protocol according to the patient's degree of aging.”

An estimation of the patient's exposure was calculated assuming that the highest number of syringes that could be used by a patient for a cycle of treatment (of a year) was seven, i.e., 7. two during the first two months and then one every two months. Accordingly, the number of patients exposed = number of syringes sold/7.

Based on this assumption, the global cumulative patient exposure has been estimated to be 42,394 patients.

With the exception of one male patient (54 years of age), all the other cases reported were female patients, aged between 40 and 63 years. No cases were reported outside the European continent.

## 3. Results

Table 2 shows the estimation on a yearly basis along with worldwide sales data; the sales (and therefore the patient exposure) almost doubled in the first and second periods and increased again by almost twice between the second and third periods. This reflects a general upward trend in the use of dermal fillers on a global scale.

All reports received were assessed from quality and safety points of view.

Routinely, IBSA Farmaceutici Italia carried out investigations and analysis of the samples or batches available to identify the possible causes of the malfunction or of the occurrence of any AEs.  Table 3 reports the list of all AEs following the injection of the medical device, with their description and evaluation from a safety perspective.

### 3.1. Quality Complaints

Overall, 18 quality complaints related to the use of the medical device were reported to IBSA Farmaceutici Italia. No adverse events or incidents were associated.

### 3.2. Safety Cases: Adverse Events

During the 3-year interval period, 12 AE reports were received by IBSA Farmaceutici Italia, as shown in Table 3.

All safety cases were received from Italy, Spain, and Germany, which were the first, third, and fourth country ranked for sales, respectively, in the period 2017-2018.

All events were described by the reporting physician as nonserious and resolved with no sequelae, generally with the use of a topical treatment (mainly corticosteroids and/or antibiotics). In one case only, the outcome was unknown due to loss to follow-up, despite the manufacturer's numerous attempts to contact the physician. No anomalies were detected after sample investigation (when available).

In 3 out of 12 reports, the medical device was not considered related to the AE description. In the other nine cases, the injection of the product was regarded as a contributory element, similar to other factors that may have likely played a role in the onset of AE, e.g., administration procedure and the patient's predisposition.

## 4. Discussion

Postmarketing safety data are very reassuring and confirm the excellent safety profile of the studied medical device resulting from its quality and manufacturing characteristics and clinical trials. It is known that the results do not reflect any of the percentages and the figures that could be described in standard clinical trials. Therefore, these data should be interpreted in this context taking into account the following points:

- First, cases reported to the manufacturer were a spontaneous initiative of the physician and/or other users; therefore, underreporting of AEs cannot be ruled out. This is plausible for mild and expected AEs and much less likely for serious or severe AEs following the awareness of the importance of reporting AEs for the interest of both patients and doctors, as this contributes to better knowledge for the medical community; moreover, in case of litigation, the reporting of AEs protects doctors
- Second, because most AEs were successfully treated by the medical doctor himself or herself and were sometimes considered to be a consequence of an inadequate injection technique, these events were not promptly reported to the Manufacturer (failure to report also occurred because certain AEs were already described in the product instructions for use);
- Finally, it is likely that in many cases, the patient is properly informed by the physician on how to act in cases of the appearance of mild and transient AEs

Hence, our results cannot be compared with those described in the context of any traditional research.

Furthermore, at the time this paper is being written, similar publications have not been identified in the context of dermo-esthetics; therefore, it is not possible to make any real comparisons with other similar products currently in use.

The decision to publish the results of this review is based on the willingness to provide doctors with all data available to improve the knowledge of this medical device.

**Figure 1:**
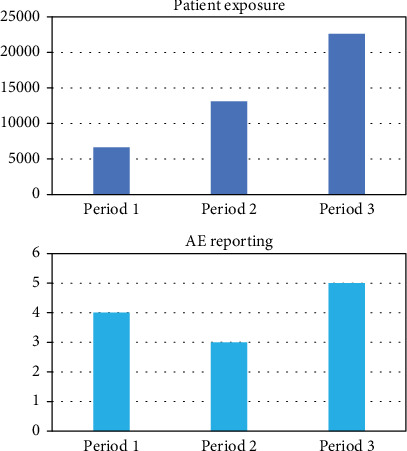
Patient exposure and AE reporting.

Despite an important increase in sales data, there is no direct proportional number of AEs reported over the 3 years considered, as shown in Figure 1.

Twelve (12) case reports were collected; many of them described the occurrence of AEs falling into the “early onset” classification; only two cases reported two late-onset adverse events.

The events have been mainly described as “swelling, edema, redness, ecchymosis, and erythema”, particularly in the malar and submalar areas, which are notoriously the most prone to show such AEs due to their peculiar anatomical features. In some other cases, the event occurred after the second injection as a probable result of a “sensitization” phenomenon that took place after the first treatment. In one case, the simultaneous administration of Botulinum Neurotoxin A was regarded as a contributing factor, even though the combination of the studied medical device and botulinum is considered suitable in particular as a treatment for wrinkles localized in the forehead and in the neck, and it has proven to be successful and well tolerated in some case reports [24]. However, all the reported cases were resolved in a short time following the recommendations that have been drawn up by panels of experts over the last few years. This has been simplified and summarized in Table 4.

Rare AEs and complications, other than those already listed in the product instructions for use, were not reported to the manufacturer.

According to the consensus statement by Philipp-Dormston et al. in 2017, preventing most AEs is possible by adhering to a series of recommendations [25]. The cosmetic surgeon should

- have a detailed knowledge of important anatomical structures and vessels of the specific target injection sites, as well as of the injectable material and its properties
- ensure formal sterile surgical preparation to prevent the introduction of bacteria and potential subsequent biofilms
- administer injections slowly and react quickly to patient pain or the occurrence of any unexpected reactions
- use aspiration if possible and a blunt cannula in high-risk zones to lower the risk of intravascular injection

On the other hand, the quality complaints reported over the 3 years were more numerous than the safety cases (18 versus 12, respectively) but always in a very limited number when compared to the number of products that have been sold worldwide. Regarding this aspect, the manufacturer identified possible causes for some of the quality complaints which received (e.g., the breakage of some of the components of the medical device during its use) a possible incorrect technique of handling by the physician, which could have emerged as a consequence of a not entirely adequate description on how to handle the components of the product. In fact, during the reference period, the manufacturer has therefore decided to improve and make clearer and more complete this section of the information leaflet to minimize these inconveniences.

Regarding published clinical data, four trials have investigated the efficacy and tolerability of the studied medical device since 2015.

(v) Laurino et al. treated 11 women with two injections of 2 mL of the product once a month for 2 months: mild AEs such as localized hematomas followed 12.1% of procedures, and they disappeared after 2-3 days [20]
(vi) Abascal and Fernandez treated 30 women with 64 mg/2 mL doses administered 30 days apart: overall, three cases of ecchymosis and three cases of pain of mild intensity were recorded; [21]
(vii) Beatini et al. evaluated 15 subjects who underwent two treatments 4 weeks apart: two cases of bruising and one case of swelling at the injection site, which resolved within 2 days, were reported [22]
(viii) Sparavigna and Tenconi monitored 64 women who were injected with two doses using the BAP (Bio Esthetic Points) technique a month apart for 16 weeks: 23% of subjects presented local minor and temporary skin reactions [23]

Safety results seem to be consistent when compared with existing data in the literature. Stojanovič et al. carried out a systematic review of published medical literature on the effectiveness and safety of different HA fillers used to enhance overall lip fullness. Twenty-two studies were included in the qualitative synthesis, with a total of 3965 subjects included. Hyaluronic acid fillers turned out to be an effective and safe treatment. The most common adverse events were local reactions at the injection sites (swelling, contusion, bruising, pain, redness, and itching), and the majority of included subjects were satisfied with the results and their physical appearance [26].

### 4.1. Study Limitations

The study was based on spontaneous reporting by the specialists who carried out the treatment. The numbers analyzed should therefore be taken with all due caution, as it is likely that not all doctors reported all the possible adverse events that their patients presented. In addition, some patients who experienced mild symptoms probably overcame them without consulting the physician. Furthermore, not all reports have detailed the patient's medical history or tracked subjects in the long term.

These elements undoubtedly represent limitations to the data reported in this study. It is reasonable to assume that there were mild symptoms that were successfully treated in a higher number of cases. Therefore, the data presented in this study are likely to be underreported [26].

## 5. Conclusions

The data presented herein validate the excellent safety characteristics of the studied medical device, which were already highlighted in the clinical trials conducted so far. However, it cannot be excluded that some AEs may not have come to the attention of the manufacturer for different reasons. The number of reports was fairly low, and there was no significant variation over the three-year period considered. Furthermore, it can be assumed that the cases, which were not reported, fell within the events listed in the product instructions for use, and therefore, it can be stated with relative certainty that use of the product did not lead to any unforeseen or serious events, since in this case, the manufacturer would have been directly involved. It is important to emphasize that the safety cases have remained steady despite the global sharp rise in product sales and consequently in the number of people exposed. In the opinion of the physicians involved and of the manufacturer, no AEs were directly and exclusively related to the administration of the product itself but rather were related to the injection technique used and some peculiar and unforeseeable characteristics related to the anatomy and physiology of the subjects. The facts that all cases had a positive outcome (successfully resolved or resolving at the moment of the report), that only one patient was lost at follow-up, and that no quality complaints resulted in any AEs give conclusive evidence of the biocompatibility, tolerability and safety of the product.

## Figures and Tables

**Table 1:** HA dermal filler AEs.

Early-onset AEs (<72 hrs)	Late-onset AEs (>72 hrs)
(i) Injection site reactions: edema, pain, erythema, itching, bleeding, ecchymosis	(i) Late infection: biofilm and fibrosis (ii) Inflammatory nodule or granuloma
(ii) Displacements: lumps, asymmetries, contour deformities	(iii) Noninflammatory nodule
(iii) Hypersensitivity reaction	(iv) Altered pigmentation (v) Scarring
(iv) Early acute infection: HSV, abscess, cellulitis	
(v) Tyndall effect	
(vi) Thromboembolism	

**Table 2:** Sales details referring to the number of syringes sold (in EU and non-EU countries) and estimated patient exposure.

Period	No. of syringes sold worldwide	No. of patients exposed
2015-2016	46,943 (42,982 EU countries; 3,961 non-EU countries)	6,706
2016-2017	91,613 (80,162 EU countries; 11,451 non-EU countries)	13,088
2017-2018	158,201 (131,548 EU countries; 26,653 non-EU countries)	22,600

**Table 3:** Detailed individual listing of AEs associated with Profhilo® 3.2% injection.

Time interval	PT demographic, country	Other suspect product	AE description & medical evaluation	Outcome
Year 1	1-F, 63 yrs, Italy	—	Edema at the injection site (zygomatic arch+suborbital area)→resulted from too hard massage after injection rather than from device itself; medical device possibly related	Topical treatment with a corticosteroid-recovering
	2-F, Italy	—	Swelling localized at the cheekbones+submalar area 20 days after the 2^nd^ injection, followed by thickening of tissue→alternative causes, i.e., depth of injection, amount of filler administered, location of the implant, process and degree of degradation of the implant, subject's predisposition & risk factors (i.e., anatomy, prior surgery, and/or filler treatment in the area); medical device possibly related	Oral and topical steroid treatment+radiofrequency+azithromycin 500 mg for 2 wks-lost to follow-up
	3-F, Italy	—	Edema of the face + periocular area after the 2^nd^ injection→sensitization after the 1^st^ injection, diagnosed as an allergic reaction by the patient herself (a nurse); the reporting physician supposed that the patient had been sensitized to the product during the first injection and therefore, she suggested the occurrence of an allergic reaction during the second injection; a medical device was possibly related	4 mg Bentelan IM-resolved
	4-F, Germany		Hypertensive crisis+vertigo+wavelike nausea 5-6 hours after the injection→histamine intolerance: the patient ate fish before treatment. A reaction reoccurred after another seafood meal, not related to the MD	Resolving

Year 2	5-F, 57 yrs, Italy		Ecchymosis at the injection site with areas of telangiectasia (with no pain, no swelling, and no dermal lesions) after the 2^nd^ injection→medical device possibly related	Topical treatment with Kelarion cream–resolved. Only few capillaries remained more visible in that area without the appearance of depressed, hardened or inflamed areas
	6-M, 54 yrs, Spain		Maculopapular erythema+itching at the injection site subsequently diagnosed as an allergic reaction-15 days after injection→pretreatment with chlorohexidine possibly causing precipitation with HA and hypersensitivity; medical device possibly related	Topical treatment with mometasone-resolved
	7-F, Germany	Botulinum neurotoxin A subcutaneous	Swelling at the injection site (lateral side of the eyes + forehead) 6 days after the injection of both products→BotToxA presumably blocked some lymphatic vessels. The medical device was not related according to both the physician and IBSA	3 ultrasound treatments-resolved

Year 3	8-F, Italy	—	Mild and transitory hematoma at the injection site→medical device possibly related	Topical treatment with arnica cream-resolving
	9-F, 59 yrs, Italy	—	Edema, intense red skin, marked hardening, mainly at the chin and malar level on the right side and nodular/granular at the cheek level→medical device possibly related	Azithromycin+deltacortene-resolved
	10-F, 40 yrs, Italy	—	Bilateral swelling more evident on the right side of the face→medical device possibly related	Ice+massage-resolved
	11-F, 54 yrs, Italy	—	Injection site reaction with redness→according to physician and IBSA, not related to Profhilo® but to patient's underlying disorder (couperose) and to the concomitant cold weather	Topical treatment with antinflammatory drug+laser-resolved
	12-F, 47 yrs, Spain		Skin eruption with redness, erythema and swelling at the injection site twice after Profhilo® injection→the physician initially considered the AE to be related to the topical application of Auriderm (vitamin K oxide) cream; however, when the same reaction occurred after the 2^nd^ injection the causal role of Profhilo® could not be ruled out	After an AE at the 2^nd^ injection: zamene 2 tbls (corticosteroid)-resolved

F: female; M: male; yrs: years; IM: intramuscular; wks: weeks; tbls: tablets.

**Table 4:** Recommendations in case of AEs occurring after dermal filler injection, according to the most recent guidelines.

AEs	Treatment
Early onset (<72 hours)	
(i) Bruising, edema, bleeding, redness, swelling	*Cold compresses, no exercise for 24* h
(ii) Tyndall effect	*Hyaluronidase*∗*and massage*
(iii) Lumping, superficial placement	*Hyaluronidase*∗*and massage*
(iv) Abscess	*Antibiotic (amoxicillin+clavulanate; cephalexin; ciprofloxacin); incision and drainage*
Late onset (>72 hours)	
(i) Displacement	*Hyaluronidase*∗
(ii) Nodule	*Hyaluronidase*∗; *antibiotic therapy (clarithromycin+moxifloxacin; ciprofloxacin; minocycline) and steroid (in case of infection); excision*
(iii) Biofilm	*Antibiotic therapy after culture*
(iv) Granuloma	*Hyaluronidase*∗; *antibiotic therapy (clarithromycin+moxifloxacin; ciprofloxacin; minocycline) and steroid (in case of infection); incision and drainage*

(Adapted from Signorini et al. 2016 and W.G. Philipp-Dormston et al. 2017). ∗From 10 to 20 U single injection up to four injection points according to the extension of the area.

## Data Availability

If requested, the safety database can be shared with editor/reviewers.
